# Effects of Music Training on Inhibitory Control and Associated Neural Networks in School-Aged Children: A Longitudinal Study

**DOI:** 10.3389/fnins.2019.01080

**Published:** 2019-10-16

**Authors:** Sarah L. Hennessy, Matthew E. Sachs, Beatriz Ilari, Assal Habibi

**Affiliations:** ^1^Brain and Creativity Institute, University of Southern California, Los Angeles, CA, United States; ^2^Thornton School of Music, University of Southern California, Los Angeles, CA, United States

**Keywords:** music, inhibitory control, executive function, longitudinal research, music training, inhibition, neuroplasticity

## Abstract

Inhibitory control, the ability to suppress an immediate dominant response, has been shown to predict academic and career success, socioemotional wellbeing, wealth, and physical health. Learning to play a musical instrument engages various sensorimotor processes and draws on cognitive capacities including inhibition and task switching. While music training has been shown to benefit cognitive and language skills, its impact on inhibitory control remains inconclusive. As part of an ongoing 5-year longitudinal study, we investigated the effects of music training on the development of inhibitory control and its neural underpinnings with a population of children (starting at age 6) from underserved communities. Children involved in music were compared with children involved in sports and children not involved in any systematic after-school program. Inhibition was measured using a delayed gratification, flanker, and Color-Word Stroop task, which was performed both inside and outside of an MRI scanner. We established that there were no pre-existing differences in cognitive capacities among the groups at the onset. In the delayed gratification task, beginning after 3 years of training, children with music training chose a larger, delayed reward in place of a smaller, immediate reward compared to the control group. In the flanker task, children in the music group, significantly improved their accuracy after 3 and 4 years of training, whereas such improvement in the sport and control group did not reach significance. There were no differences among the groups on behavioral measures of Color-Word Stroop task at any time point. As for differences in brain function, we have previously reported that after 2 years, children with music training showed significantly greater bilateral activation in the pre-SMA/SMA, ACC, IFG, and insula during the Color-Word Stroop task compared to the control group, but not compared to the sports group (Sachs et al., 2017). However, after 4 years, we report here that differences in brain activity related to the Color-Word Stroop task between musicians and the other groups is only observed in the right IFG. The results suggest that systematic extracurricular programs, particularly music-based training, may accelerate development of inhibitory control and related brain networks earlier in childhood.

## Introduction

Executive functions are broadly defined as top–down processes related to goal acquisition and attention that primarily recruit the brain’s prefrontal network (Miller and Cohen, 2001). Inhibitory control, a sub-construct of executive function (Miyake et al., 2000), refers to the ability to suppress a primary response. It is correlated with the activation in the dorsolateral prefrontal cortex (DLPFC), anterior cingulate cortex (ACC), supplementary and pre-supplementary motor cortex (SMA/pre-SMA), insula, and inferior frontal gyrus (IFG) (Niendam et al., 2012). Inhibitory control has been shown to be predictive of academic success (Mischel et al., 1989; Alloway et al., 2005; Duckworth and Seligman, 2005; Kirby et al., 2005), career success (Bailey, 2007), positive socioemotional wellbeing (Hughes and Dunn, 1998; Lengua, 2003; Eisenberg et al., 2005), wealth (Moffitt et al., 2011), reduced substance abuse risk and incarceration (Moffitt et al., 2011), and physical health (Seeyave et al., 2009; Miller et al., 2011).

The evidence supporting the association between inhibitory control and positive life outcomes has encouraged the development of educational programs aimed at improving these skills in childhood. Several studies observed enhancement of inhibitory control after short-term exercise programs among children who were overweight (Davis et al., 2011), who were diagnosed with attention deficit hyperactivity disorder (Chang et al., 2012), as well as among typically developing, non-overweight children (Chen et al., 2014). Similar improvements have been reported in children involved in martial arts (Lakes and Hoyt, 2004), mindfulness training (Flook et al., 2010), and classroom-based programs such as Tools of Mind (Diamond et al., 2007). In addition to behavioral improvements, these interventions produced training-related activation increases in regions of the cognitive control network changes in the cognitive control network in both children (Davis et al., 2011; Voss et al., 2011) and adults (Allen et al., 2012; Berkman et al., 2014). Others cite neural activation decreases associated with increased efficiency of inhibitory control after an intervention (Chaddock-Heyman et al., 2013; Nishiguchi et al., 2015).

Recently, there has been increasing interest in the impact of music interventions on developing inhibition control. Playing a musical instrument requires a variety of functions, including coordination of fine motor skills and different streams of auditory input (Zatorre et al., 2007), the rapid adjustment of one’s motor behavior in response to mistakes (Jentzsch et al., 2014). To meet the technical demands of playing their instrument, musicians must continue to play as they inhibit attention away from one hand to focus on a different movement in the other hand. Like any practice-dependent activity or acquiring any skill, playing a musical instrument requires focused attention and self-discipline, prioritizing practice over other, more instantly gratifying, activities. This is particularly important in a group setting, where there are many social distractors, such as side conversations or other playing a piece incorrectly, that musicians must ignore for the benefit of the performance. For these reasons, musicians have been often cited as a model from which to investigate neuroplasticity (Münte et al., 2002).

In spite of this, contributions of musical abilities on inhibition in adults are not clear. While some studies have found that professional adult musicians show faster reaction times on a Color Stroop Task than amateur adult musicians (Travis et al., 2011) and untrained individuals (Bialystok and Depape, 2009), and faster reaction times than untrained individuals on a visual Simon task (Bialystok and Depape, 2009), Zuk et al. (2014) did not find differences in response times between adult musicians and untrained individuals on a Color Stroop Task, and Slevc et al. (2016) found music training to be unrelated to scores in an Auditory Stroop and Simon Arrows task.

Studies with children are similarly inconclusive. In a Simon task, children aged 10–11 who were musically trained showed reduced reaction time differences between congruent and incongruent trials when compared to children without music training (Joret et al., 2017). When asked to name shapes and arrows in an opposite manner (e.g., ‘up’ when presented with a ‘down’ arrow), musically trained children aged 3–9 performed significantly faster than musically un-trained children (Saarikivi et al., 2016). In another study, Holochwost et al. (2017) reported improvements in go-no-go task accuracy in 7–13-year-old children participating in an *El Sistema* orchestral program after 2 years and again after 3 years of training. Children aged 5–7 involved in school music education had greater increases in scores on a go-no-go task over two and a half years when compared to a visual arts and passive control group (Jaschke et al., 2018). Similarly, 4–6-year-old children involved in a music listening program performed better than those in an arts training program on go-no-go accuracy at post-test (Moreno et al., 2011). In an fMRI investigation of cognitive control networks, Zuk et al. (2014), demonstrated that musically trained children aged 9–12 had greater activation in the pre-SMA/SMA and right VLFC during a set shifting task as compared to non-musician children. Our group recently reported that, children aged 8–9 with 4 years of music training, as compared to children without any systematic training, had greater BOLD signal during incongruent trials of a color-word Stroop task in inhibition-related neural regions, including the pre-SMA/SMA, precentral sulcus, ACC and IFG (Sachs et al., 2017). However, no significant differences were observed between musically trained children and children involved in sports training. Additionally, neither our group (Sachs et al., 2017), nor Zuk et al. (2014) observed behavioral differences between groups, and others have reported no significant benefits of music training on inhibition (Schellenberg, 2011), leaving the findings inconclusive.

Several factors possibly contribute to the lack of consistency in these findings, including the absence of an active comparison group in some studies, and a great degree of variation in the music training programs studied. For example, Moreno et al. (2011) used a computerized curriculum with a focus on listening rather than learning to play a musical instrument, which involves sensory-motor learning and participation in a group setting (e.g., Holochwost et al., 2017). Even among studies that agree on the definition of “music training” as instrumental instruction, many differ on the length of training required to label participants as “musicians” or “non-musicians.” In Schellenberg (2011) study, child musicians were described as having at least 2 years of music lessons outside of school; whereas, children in Zuk et al. (2014) study had been playing for an average of 5 years, while Degé and Schwarzer (2011) defined music training as a continuous variable. These differences are of particular importance due to the rapid development of inhibitory control during early to middle childhood, in which one or 2 years of regular participation in a music program could provide significant improvements in skill performance. Differences in individual and group music training between studies may also affect comparability between studies, since the social aspect of music making requires more integration of cognitive and social functions than solo playing (Keller, 2008). Playing in an ensemble where one is required to follow a conductor’s command, to attend to other members of the ensemble, and to adjust behaviors in response to other players, could improve inhibition skills at a faster rate than individual musical practice.

Finally, a limitation of current research in the field is the difficulty in recruiting a study sample that reflects society’s growing socio-economic status (SES) and cultural diversity. While a few groups (e.g., Holochwost et al., 2017) have investigated underrepresented populations, many studies are conducted with participants from what Henrich et al. (2010) called WEIRD societies (white, English speaking, industrial, and from rich and democratic countries), and may be generalizable only to a narrow portion of society at large. To draw broader conclusions about the impact of music training throughout society, more research involving participants from diverse backgrounds is needed.

In the present study, we investigate the effects of music training on inhibitory control using behavioral and neuroimaging methods, aiming to extend the previously reported findings (Sachs et al., 2017). Over the course of 4 years, we compare children involved in music training with children involved either in sports training or no systematic enrichment training. By conducting this investigation longitudinally, and implementing a pre-training baseline assessment, we attempt to differentiate effects of training from pre-existing biological contributions. To our knowledge, this is the first longitudinal study using neuroimaging to specifically assess inhibitory control in children involved in music training. Additionally, by comparing children involved in music training to children involved in sports training, we assess whether any effects observed in measures of inhibition are related to music training specifically or are associated with any type of extra-curricular activity that is socially engaging and motivating.

We hypothesize: (a) that children involved in music training will show greater improvements on behavioral measurements of inhibition than will children involved in no systematic training and children involved in sports training, evidenced by reduced reaction time and improved accuracy; (b) that, during an fMRI inhibition task, children involved in music training as compared to children with no systematic training will show greater activation of brain regions associated with inhibitory control, including the IFG, SMA/pre-SMA, ACC, and insula, continuing the pattern reported after 2 years of training (Sachs et al., 2017).

## Materials and Methods

### Participants

Data for this report were collected as part of an ongoing longitudinal study investigating the effects of music training on child brain, cognitive, and socioemotional development (Habibi et al., 2014). Eighty-eight participants (36 female, mean age = 6.81 years, *SD* = 0.69) were recruited from community music and sports programs, and public elementary schools in the Greater Los Angeles Area. Participants were from three groups: 28 children (11 female) who had enrolled and were about to begin participation in the Youth Orchestra of Los Angeles at the Heart of Los Angeles program (hereafter called “music group”), 29 children (12 female) who had enrolled and were about to begin participation in community based soccer or swimming training formed the first comparison group (hereafter called “sports group”), 31 children (13 female) recruited from public elementary schools in the same Los Angeles Area who, at the time of recruitment, were not engaged in an organized and systematic after-school music or sports programs formed the second comparison group (hereafter called “control group”).

#### The Music Training Program

The Youth Orchestra of Los Angeles at Heart of Los Angeles (YOLA at HOLA) is inspired by the Venezuelan approach known as “El Sistema,” offering free group-based music instruction 4–5 days a week to children from underserved communities of Los Angeles. The program emphasizes systematic, high intensity group music training, focusing on rhythm, melody, harmony, and ensemble practice with a goal of promoting social inclusion. Children (up to 20 per year) are selected by lottery from a list of interested families and, after selection, provided with a violin or viola. The curriculum includes group stringed instrument practice, group singing, Orff, and musicianship (ear training and theory skills), totaling 6–7 h of music instruction per week. Individual practice at home is left to discretion of the families.

#### The Sports Training Program

The soccer and swimming programs offer free or low-cost training in a community setting to all children whose parents choose to enroll. The soccer program consisted of a 2-h practice three times a week, and a 1-h game each weekend. Soccer practices included warmups, team cheers, skill training (dribbling, passing, etc.), and simulated games. The swimming program consisted of a 1-h practice, two times a week, with additional recreational sessions each weekend. Swimming practices included fitness, endurance, water safety, and stroke development. Both programs were taught by professional coaches.

The sports training group was selected as a comparison group to control for aspects of musical training that would likely be shared by those in a regular, extra-curricular activity such as social engagement, discipline, and sustained effort. Additionally, sports training was chosen due to its sensory motor learning, a component shared with music training. These aspects alone may have beneficial effects on development of both cognitive and social skills, and thus including an active comparison group is essential.

### Exclusion Criteria

Children were excluded from the study if they had a history of psychiatric or neurological disease. At all assessment times, participants’ parents were interviewed to ensure that their children had not been diagnosed with a developmental or neurological disorder in the previous year.

During the first visit of each year, children were asked to report any activities they had begun to participate in outside of school. Participants in the control group were excluded from analyses if they had been involved in an extra-curricular music or athletic activity three times a week for at least 6 months. Participants in the sports group were excluded from analyses if they had been involved in a music training program three times a week for at least 6 months. Participants in the music group were excluded from analyses if they had discontinued participation in the music program.

**FIGURE 1:**
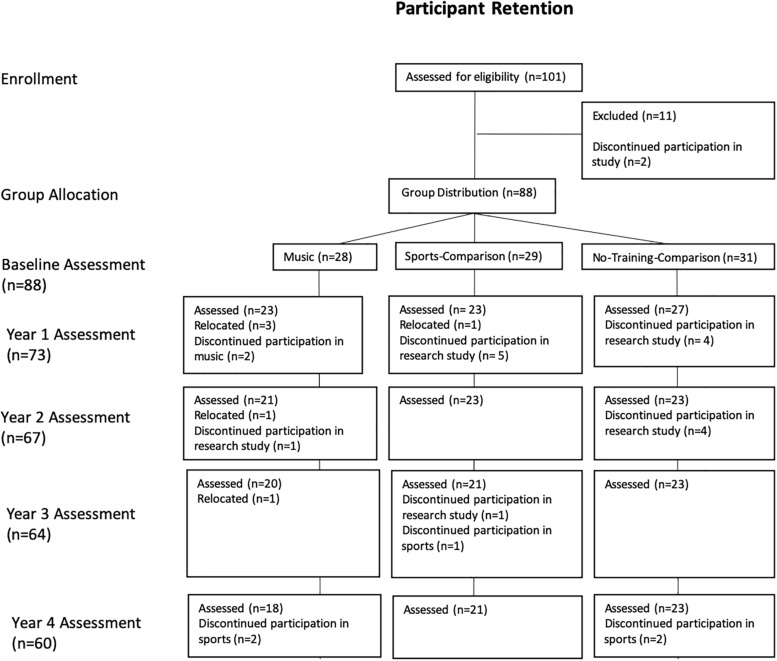
Participant retention between groups from baseline to Year 4.

Five time points were used to assess the development of inhibition (Figure 1). Children were assessed annually, at approximately the same time each year. Hereafter, “baseline” will be used to refer to the initial assessment, prior to the start of any training (in case of music and sports and no training in case of the control), and subsequent testing times will be referred to as “Year 1” through “Year 4.”

### Socio-Economic Status

All participants came from equally underprivileged communities in Los Angeles (Habibi et al., 2014). Socioeconomic status (SES) was assessed through parental interviews conducted by research assistants who were native speakers of the parents’ preferred language (i.e., English, Spanish, or Korean). Parent interviews included questions ascertaining maternal and paternal education and occupation and annual family household income and size. An SES score was calculated as the mean of each parent’s education and annual income. Education level was scored on a 5-point scale: (1) elementary/middle school; (2) high school; (3) college education; (4) master’s degree (MA, MS, MBA); (5) professional degree (Ph.D., MD, JD). Annual income was also scored on a 5-point scale: (0) <$10,000; (1) $10,000–$19,999; (2) $20,000–$29,000; (3) $30,000–$39,999; (4) $40,000–$49,999; (5) >$50,000.

The ethnic distribution included children of Latino, Korean, and African-American backgrounds. 97.7% of participants were bilingual, raised in English–Spanish (93.2%) or English–Korean (4.5%) speaking households while attending English speaking schools that did not offer systematic music programs for their students.

### Procedures

Recruitment and induction protocols were approved by the University of Southern California Institutional Review Board. Informed consent was obtained in writing from the parents/guardians in the preferred language on behalf of the child participants, and verbal assent, at each year, was obtained from each child. Either the guardians or the children could end their participation at any time. Participants (parents/guardians) received monetary compensation ($15 per hour) for their child’s participation and children were awarded small prizes (e.g., toys or stickers). To recognize continuous participation, participants were given a $50 bonus after each year’s testing and sent small holiday and birthday gifts. All children were tested individually at the Brain and Creativity Institute at the University of Southern California, or at Heart of Los Angeles in a designated private room.

### Behavioral Assessment

**FIGURE 2:**
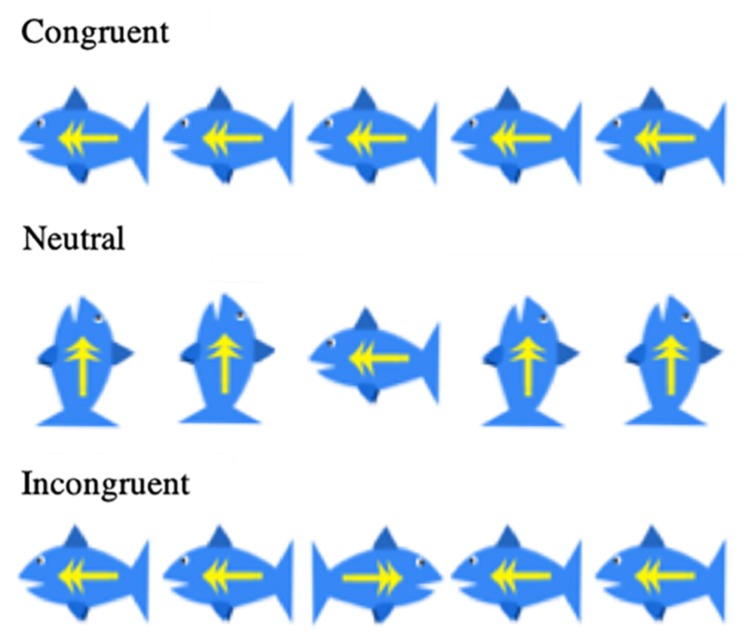
Flanker fish task conditions.

Cognitive development was assessed using the Wechsler Abbreviated Scale of Intelligences (WASI-II) for children (Wechsler, 2011). Cognitive inhibition was measured with a child-friendly version of the Flanker task (Davidson et al., 2006; Diamond et al., 2007) (Figure 2). An Animal Stroop task was used at Year 1 (Wright et al., 2003), and a computerized Color-Word Stroop task was used starting Year 2. Additionally, we measured inhibitory control using a version of the delayed gratification task (Mischel et al., 1989), where children were presented with increasing reward sizes that they could elect to take immediately, or wait in favor of a larger reward at a future time.

See Supplementary Material for a detailed description of each task.

### Neuroimaging Assessment

Children underwent anatomical, diffusion, and functional MR imaging of their brain. A child-friendly protocol was implemented that included a training session prior to scanning, where children learned about the scanner and familiarized themselves with the environment in a mock scanner. Results from structural and diffusion scans are discussed in a previous report (Habibi et al., 2017).

**FIGURE 3:**
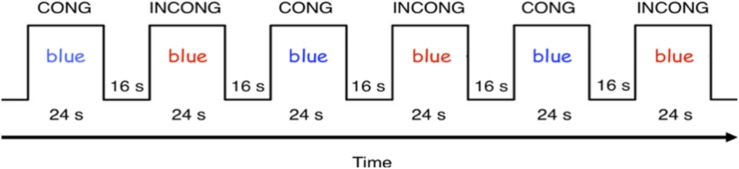
fMRI Color-Word Stroop task paradigm.

The fMRI consisted of a modified version of the Color-Word Stroop task, designed for performance inside the fMRI scanner (Figure 3), and performed at Years 2 and 4 (see Supplementary Material for detail). Children completed two functional runs, of six blocks each. Blocks were divided between word and color congruent, and incongruent conditions. Each block was followed by a 16 s rest period for a total scan time of 240 s (120 TRs).

A 3T MAGNETOM Prisma System was used to acquire high-resolution T1-weighted structural MRI images, using a 20-channel head coil (1 mm × 1 mm × 1 mm resolution over a 256 × 256 mm × 256 mm FOV; T1/TE/TR = 850/32.05/2300 ms; flip angle = 8°; GRAPPA acceleration factor *R* = 2). A gradient echo, echo-planar, T2^∗^-weighted pulse sequence was used to acquire functional images (TR = 2000 ms, one shot per repetition, TE = 25 ms, flip angle = 90°, 64 × 64 in-plane resolution). Forty-one slices covering the entire brain were acquired (3 mm × 3 mm × 3mm voxel resolution). Each run of the task consisted of 165 volumes.

### Analysis

Statistical analyses were performed using R statistics (R Core Team, 2018). Only participants who had completed all time-point assessments of a given task were included in the final analysis. Outliers, defined as scores two or more standard deviations above or below the mean, were removed. Not all tasks were performed every year (Table 1). When sphericity assumptions were violated, degrees of freedom were corrected using Greenhouse–Geisser epsilon adjustments. The alpha-level used in all analyses is 0.05.

**TABLE 1:** Task completion per year.

**Task**	**Baseline**	**Year 1**	**Year 2**	**Year 3**	**Year 4**
Animal Stroop		□			
Color Stroop		□	□	□	□
Delayed Gratification				□	□
Flanker Fish			□	□	□
WASI	□	□	□	□	□
fMRI Stroop			□		□

#### Behavioral Analysis

Separate repeated measures ANOVAs were conducted for each task, with group as the between-groups factor and year as the within-groups factor. For tasks that included multiple conditions (e.g., reward size or congruency), condition was included as an additional within-groups factor. For tasks that included multiple variables (e.g., accuracy and reaction time), separate repeated measures ANOVAs were used for each variable.

The animal Stroop task, completed at Year 1, was analyzed with one-way ANOVAs for each reaction time and errors, obtained by subtracting congruent trials from incongruent trials. For the color-word Stroop task, participants responded verbally Years 2 and 3, and through keypresses Year 4, thus analyses for Year 2 and 3 were conducted separately from Year 4. For Year 2 and 3, a repeated measures ANOVA was conducted for accuracy and reaction time, with group as the between-groups factor, and year and congruency as within-groups factors. For Year 4, one-way ANOVAs were conducted for accuracy and reaction time. Stroop reaction time performed during fMRI was analyzed with a repeated measures ANOVA, with between-subjects factors of congruency and year and group as the within-subjects factor. *Post hoc* tests for all tasks were computed using Tukey’s HSD, and Cohen’s *d* effect size was calculated and reported.

#### fMRI Analysis

##### Whole brain

All analyses of functional MR data were completed using the FMRIB Software Library (FSL). Pre-processing included removal of non-brain tissue (BET), motion correction (MCFLIRT), slice-timing correction, spatial smoothing (5.0 mm FWHM Gaussian kernel), and high pass temporal filtering (140 s). Motion scrubbing was conducted for each functional run to correct for additional head motion, using root-mean-squares intensity differences (dvars) to determine which slices should be regressed out during GLM analysis (Power et al., 2012). Slices with dvars values greater than the 75th percentile +1.5^∗^inter-quartile range were included in a confound matrix added to the GLM. A fixed effects analysis was used to combine the two functional runs for each participant, and FLIRT was used to register images to a high-resolution structural and standard space with 12 DOF and a 2-mm MNI template. The task was modeled with a regressor for each congruency condition using a boxcar convolved with a double-gamma hemodynamic response function. BOLD signal between the task conditions (congruent, incongruent) were contrasted using a general linear model. Models were combined into a mixed-effects analysis, and independent two-sample *t*-tests were used to determine brain activation differences during these contrasts between groups. A repeated measures ANOVA was used to compare contrasts between groups across years. Age at the time of scan was used as a covariate of non-interest. Statistical inference was completed using Z images and FSL’s cluster thresholding, using a cutoff of Z > 2.3, and a cluster size probability of *p* = 0.05.

##### Region of interest

Region of interest (ROI) analysis was conducted to assess percent signal change in regions selected *a priori* due to their known involvement in cognitive control mechanisms (pre-SMA/SMA, ACC, and IFG). 8-voxel spheres were drawn with center coordinates located at the peak voxel base on significant clusters found in the group-level all subject, incongruent > congruent contrast from Year 2 results (Sachs et al., 2017). Percent signal change was calculated from beta values using Featquery in FSL. A repeated measures ANOVA was conducted for each ROI, with year as the within-subjects factor, and group as the between-subjects factor.

## Results

A chi-squared test revealed no significant group differences in sex [χ^2^ (2, *N* = 53) = 2.54, *p* > 0.05]. Age at baseline was higher in the Control group (*M* = 7.04 years, *SD* = 0.49 years) than in the Music group (*M* = 6.48 years, *SD* = 0.42 years), [*F*(2,52) = 5.34, *p* < 0.01]. There were no differences between the sports group age (*M* = 6.69 years, *SD* = 0.75 years) and any other group (*p* > 0.05), and there were no differences between groups in age at any other year. The number of included subjects differed between tasks, thus age at baseline was used as a co-variate only in cases in which there was a significant group difference at onset in subjects that completed all years of a given task. There were no differences between the groups in gender, SES, or cognitive abilities at baseline assessment (Habibi et al., 2014) and thus these factors were not included in the analysis.

### Behavioral Results

#### WASI-II

The WASI-II was completed at all five assessment times by 52 participants (Music *n* = 17, Sport *n* = 17, Control *N* = 18). For FSIQ-4, no main effect of group (*p* > 0.05) or year (*p* > 0.05) was observed. No year by group interaction effect was found (*p* > 0.05). For PRI, no main effect of group was observed (*p* > 0.05). A main effect of year was observed [*F*(4,196) = 2.65, *p* < 0.05, η^2^ = 0.01], but *post hoc* comparisons did not reach significance (*p* > 0.05). No year by group interaction was observed (*p* > 0.05). No effects of group, year, or year by group interactions were observed in VCI (all *p* > 0.05), or FSIQ-II (all *p* > 0.05).

#### Stroop

The animal Stroop task was completed at Year 1 by 54 participants (Music *N* = 19, Sport *N* = 14, Control *N* = 21). No significant group differences were observed (*p* > 0.05). The color-word Stroop task was assessed at Year 2, Year 3, and Year 4, and completed by 43 participants (Music *N* = 17, Sport *N* = 12, Control *N* = 14). For Year 2 and Year 3, a significant main effect of year on accuracy was found [*F*(1,39) = 11.96, *p* < 0.01, η^2^ = 0.24], where accuracy was greater Year 3 (*M* = 0.96, *SD* = 0.06) than Year 2 (*M* = 0.94, *SD* = 0.08) (*p* < 0.01, *d* = 0.40). A significant main effect of condition was also observed [*F*(1,39) = 36.74, *p* < 0.001, η^2^ = 0.49], where accuracy was greater on the congruent condition (*M* = 0.99, *SD* = 0.03) than the incongruent condition (*M* = 0.92, *SD* = 0.08) (*p* < 0.001, *d* = 1.12). No main effect of group was observed (*p* > 0.05). No year by group (*p* > 0.05) or condition by group (*p* > 0.05) interaction was observed. No significant year by condition by group interaction effect was found (*p* > 0.05). For reaction time (incongruent minus congruent trials), no main effect of year (*p* > 0.05) or group (*p* > 0.05) or year by group interaction (*p* > 0.05) was observed.

For Year 4, a main effect of condition on accuracy was observed in a one-way ANOVA [*F*(1,40) = 22.27, *p* < 0.001, η^2^ = 0.36], where congruent trials (*M* = 0.86, *SD* = 0.15) were responded with more accuracy than incongruent trials (*M* = 0.80, *SD* = 0.16) (*p* < 0.001, *d* = 0.37). There was no main effect of group (*p* > 0.05), or an interaction effect between condition and group (*p* > 0.05). For reaction time (incongruent minus congruent trials), no main effect of group was found (*p* > 0.05).

#### Flanker Fish Task

**FIGURE 4:**
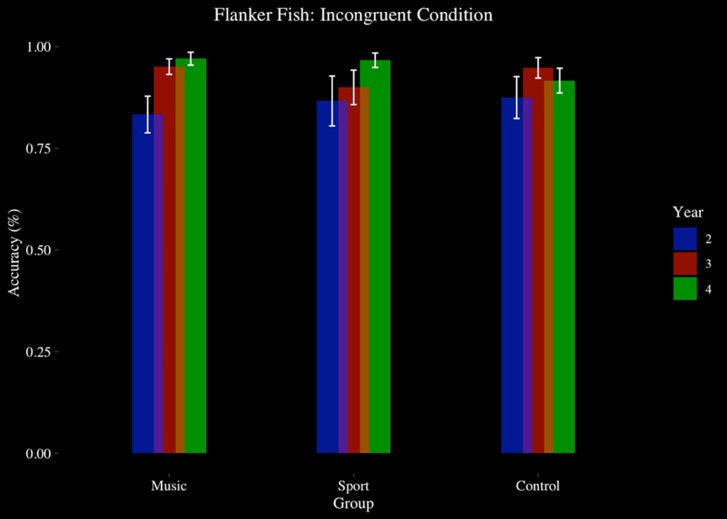
Flanker fish task, accuracy, by group and year: incongruent condition.

The flanker task was completed by 48 participants at Year 2, Year 3, and Year 4 (Music *N* = 17, Sport *N* = 15, Control *N* = 16). There was a significant main effect of year on accuracy [*F*(1.17,52.68) = 7.02, *p* < 0.01, η^2^ = 0.14], in which participants improved their accuracy each year. There was a significant main effect of condition [*F*(1.54,69.45) = 13.58, *p* < 0.001, η^2^ = 0.23], where participants responded more accurately on congruent trials (*M* = 0.97, *SD* = 0.12) than incongruent trials (*M* = 0.91, *SD* = 0.15) (*p* < 0.001, *d* = 0.42) and more accurately on neutral trials (*M* = 0.96, *SD* = 0.13) than incongruent trials (*p* < 0.001, *d* = 0.30). There was no main effect of group (*p* > 0.05), and no interaction effect of condition by group (*p* > 0.05), or year by group (*p* > 0.05). There was no significant year by condition interaction effect (*p* > 0.05). There was a significant year by condition by group interaction effect [*F*(8,180) = 2.061, *p* < 0.05, η^2^ = 0.08]. *Post hoc* analysis revealed that the condition by year interaction effect was significant in the music group only, [*F*(4,64) 4.67, *p* < 0.01, η^2^ = 0.06], where during incongruent trials participants responded with higher accuracy at Year 4 (*M* = 0.97, *SD* = 0.07) (*p* < 0.01, *d* = 0.98) and Year 3 (*M* = 0.95, *SD* = 0.08) (*p* < 0.05, *d* = 0.82) compared to Year 2 (*M* = 0.83, *SD* = 0.19). No such year by condition effect was observed in the sport (*p* > 0.05) or control groups (*p* > 0.05) (see Figure 4).

**FIGURE 5:**
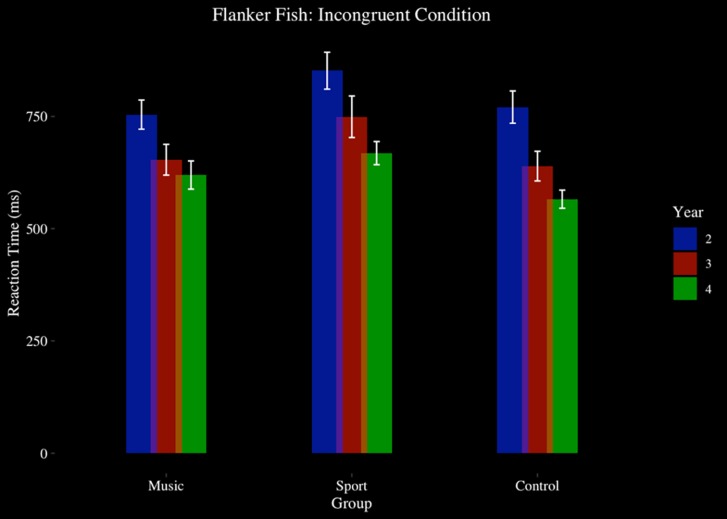
Flanker fish task, reaction time, by group and year: incongruent condition.

For reaction time, there was a significant main effect of year [*F*(2,90) = 41.43, *p* > 0.001, η^2^ = 0.48]. A main effect of group approached significance [*F*(2,45) = 2.98, *p* = 0.06, η^2^ = 0.12], indicating that the Sport (*M* = 680.64 ms, *SD* = 151.02 ms) trended toward longer reaction times than Music (*M* = 619.83 ms, *SD* = 138.76 ms) (*p* < 0.001, *d* = 0.42) and Control (*M* = 602.77 ms, *SD* = 137.37 ms) (*p* < 0.001, *d* = 0.54). There was a significant main effect of condition on reaction time [*F*(1.77,79.47) = 189.40, *p* < 0.001, η^2^ = 0.81]. No year by group interaction effect was observed (*p* > 0.05). A condition by group interaction effect approached significance [*F*(4,90) = 2.41, *p* = 0.06, η^2^ = 0.10], indicating a trend that group differences were significant in the incongruent and congruent conditions, and not in the neutral condition. No year by condition by group interaction effect was observed (*p* > 0.05) (see Figure 5).

#### Delayed Gratification

The delayed gratification task was assessed at Year 3 and Year 4, completed by 59 participants (Music *n* = 18, Sport *n* = 21, Control *n* = 20). For quarters, no main effect of year (*p* > 0.05), or group was observed (*p* > 0.05). There was a significant main effect of reward size [*F*(1.72,96.2) = 4.88, *p* < 0.05, η^2^ = 0.08], where large rewards (*M* = 0.89, *SD* = 0.32) were saved more than small rewards (*M* = 0.77, *SD* = 0.42) (*p* < 0.05, *d* = 0.32). No year by group (*p* > 0.05) or reward size by group (*p* > 0.05) interaction was observed. No year by reward size by group interaction was observed (*p* > 0.05). Although group differences were non-significant, the music group consistently saved 100% of quarters in the large condition both Year 3 and Year 4, while the Sport and Control groups saw a decline in large reward delay, from Year 3 to Year 4.

**FIGURE 6:**
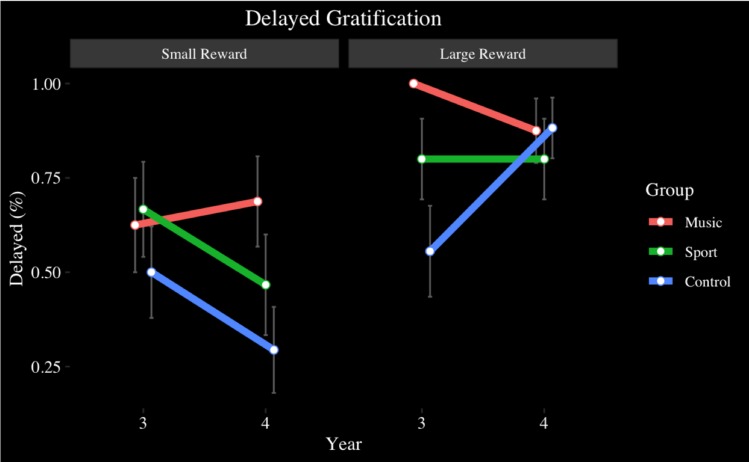
Delayed Gratification task, candies, by group and year: small and large size conditions.

For M&Ms, no main effects of year (*p* > 0.05), or group (*p* > 0.05) were observed. A main effect of reward size [*F*(2,112) = 15.42, *p* < 0.001, η^2^ = 0.22], where large (*M* = 0.81, *SD* = 0.39) (*p* < 0.001, *d* = 0.62) and medium rewards (*M* = 0.76, *SD* = 0.43) (*p* < 0.01, *d* = 0.49) were saved more than small rewards (*M* = 0.54, *SD* = 0.50). No year by group (*p* > 0.05) or reward size by group (*p* > 0.05) was found. No year by reward size interaction effect was observed (*p* > 0.05). A year by reward size by group interaction was found [*F*(3.57,99.81) = 2.80, *p* < 0.05, η^2^ = 0.09]. Follow up analyses indicated that the year by group interaction was significant only in the large reward condition [*F*(2,56) = 3.49, *p* < 0.05, η^2^ = 0.11], where the Music group (*M* = 1.00, *SD* = 0.00) saved more M&Ms than the Control group (*M* = 0.56, *SD* = 0.51), (*p* < 0.01, *d* = 1.19) at Year 3 (see Figure 6). A non-significant trend indicated a stepwise increase in reward delay from small to large rewards that was present only in the Music group, but not the Sport or Control groups, where the music participants saved more large rewards than medium rewards and more medium rewards than small rewards. At Year 4, the group by reward size interaction was no longer significant.

### fMRI Results

The fMRI protocol was completed by 40 participants at Year 2 and Year 4 (Music *N* = 14, Sport *N* = 11, Control *N* = 15).

#### Reaction Time

During the fMRI Stroop task, a main effect of condition on reaction time was observed [*F*(1,26) = 99.26, *p* < 0.001, η^2^ = 0.22], where participants performed faster at congruent trials (*M* = 654.72 ms, *SD* = 129.21 ms) than incongruent trials (*M* = 810.51 ms, *SD* = 169.56 ms) across years. There was no main effect of group or year (*p* > 0.05), and no interaction between group, year, or condition (*p* > 0.05).

#### Whole Brain

**FIGURE 7:**
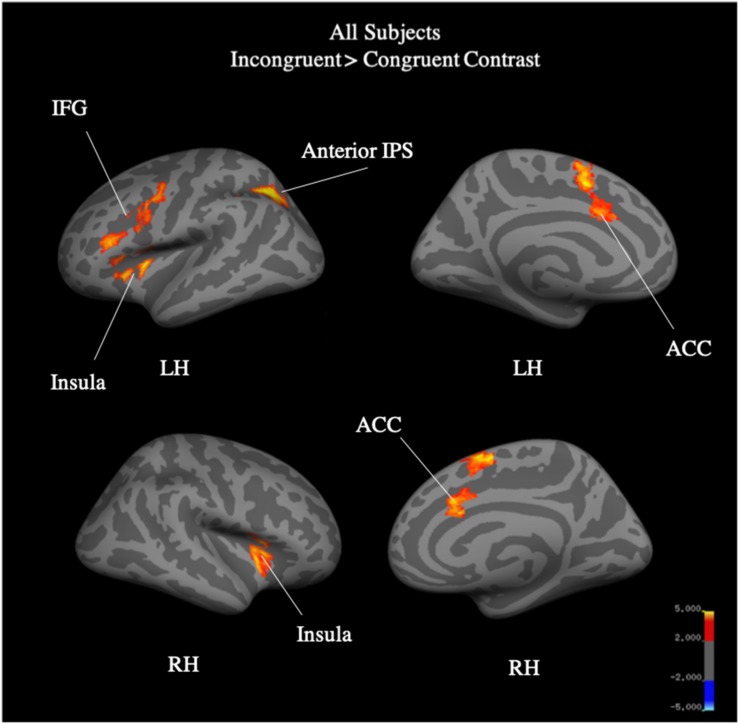
All-subject, whole brain activation for incongruent > congruent contrast of fMRI Color-Word Stroop task at Year 4.

**FIGURE 8:**
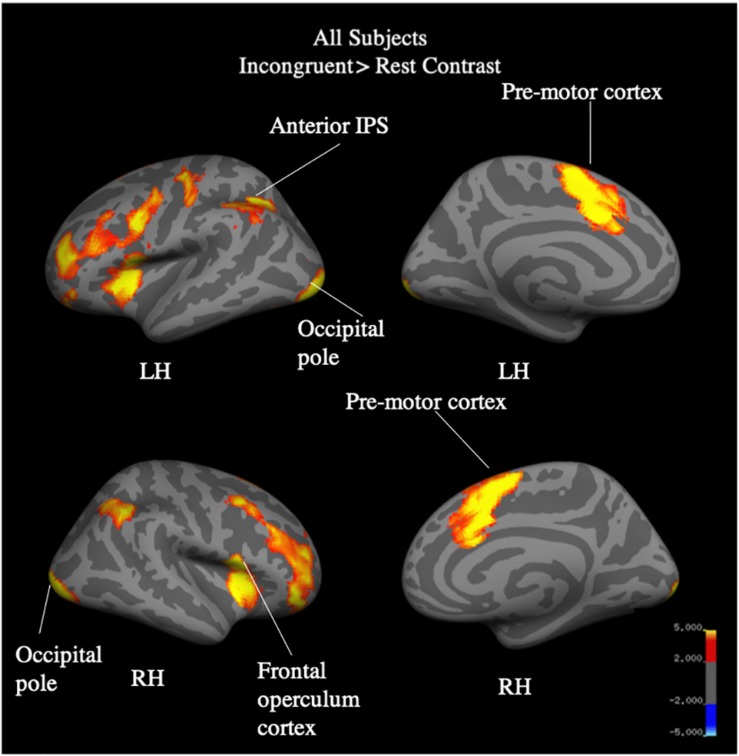
All-subject, whole brain activation for incongruent > rest contrast of fMRI Color-Word Stroop task at Year 4.

**FIGURE 9:**
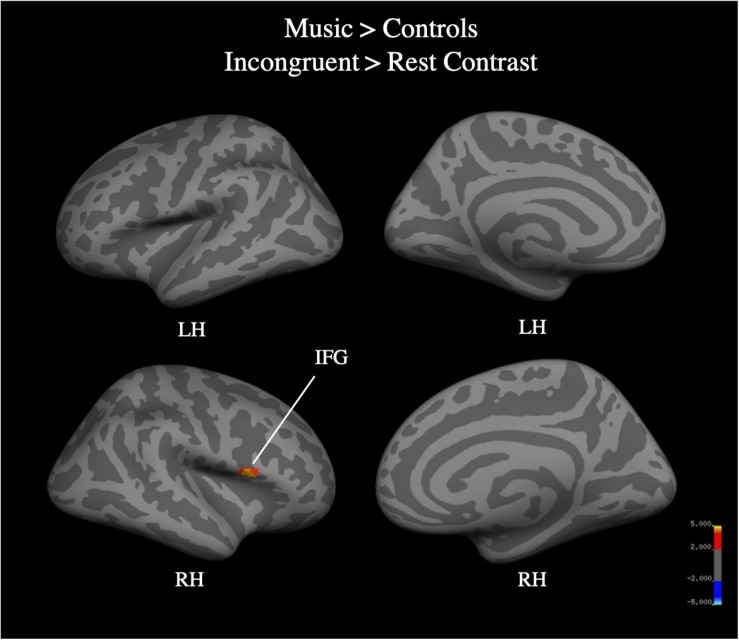
Music group > control group, whole brain activation for incongruent > rest contrast of the fMRI Color-Word Stroop task at Year 4.

Whole brain analysis of the incongruent > congruent condition between Year 2 and Year 4 revealed no effect of year and no interaction between year and group on BOLD signal. Due to the small number of participants (*N* = 25) who were scanned in both Year 2 and Year 4, we additionally analyzed Year 4 (*N* = 40) separately. Whole-brain analysis for the incongruent > congruent condition contrast revealed significant signal differences across groups in the left IFG, bilateral ACC, bilateral insula, and the left anterior intra-parietal sulcus at Year 4 (see Figure 7). There were no significant differences in BOLD signal found between groups. Analysis of the incongruent > rest condition revealed significant signal differences across groups in the bilateral premotor cortex, right frontal operculum cortex (Broca’s area), bilateral occipital pole, left anterior intra-parietal sulcus, and left angular gyrus (see Figure 8). Musicians compared to the control group showed significantly greater activation of the right inferior frontal gyrus in this contrast (see Figure 9). No other group differences in BOLD signal were observed at Year 4 (see Table 2).

**TABLE 2:** Significant clusters for the fMRI Color-Word Stroop task at Year 4.

**Contrast**	**Group**	***z*-score**	***x***	***y***	***z***	**Hemisphere**	**Region**
Incongruent > Congruent	All	5.92	–50	30	20	L	IFG, pars triangularis
		5.27	10	18	30	R	ACC
		5.66	–28	–60	42	L	Anterior intra-parietal s
		5.96	40	12	0	R	Insula
Incongruent > Rest	All	7.64	–2	4	58	L/R	Premotor Cortex
		7.62	46	12	2	R	Frontal Operculum Cortex
		8.45	18	–98	–8	R	Occipital Pole
		8.59	–24	–98	–12	L	Occipital Pole
		5.79	–32	–54	38	L	Anterior intra-parietal s
		5.36	50	–50	56	R	Angular Gyrus
	Music > Control	5.08	50	14	6	R	IFG, pars opercularis

* Coordinates represent the peak voxel of the cluster in MNI space.*

#### Region of Interest

**FIGURE 10:**
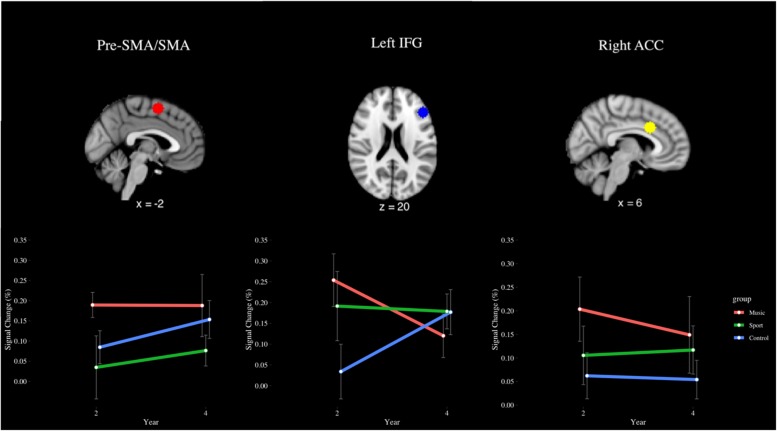
Percent signal change in the pre-SMA/SMA, left IFG, and right ACC during the incongruent > congruent contrast of the fMRI Color-Word Stroop Task, by group and year.

Region of interest analysis of the incongruent > congruent contrast (see Figure 10) in the SMA revealed an effect of group that approached significance (*p* = 0.06), where the music group trended toward greater signal change (*M* = 0.19%) than the sport group (*M* = 0.06%) across years. In the left IFG, a year by group interaction effect approached significance (*p* = 0.09), indicating that the music group (*M* = 0.25%) trended toward greater signal change than the control group (0.19%) year 2, but not year 4 (music *M* = 0.12%, sport *M* = 0.19%). In the right ACC, a significant main effect of group was observed [*F*(2,28) = 3.66, *p* < 0.05, η^2^ = 0.07], where the music group (*M* = 0.18%) had greater percent signal change than the control group (*M* = 0.06%), but this difference was not significant after correcting for multiple comparisons (*p* = 0.07, *d* = 0.61). No other significant main or interaction effects were observed for this contrast (*p* > 0.05).

**FIGURE 11:**
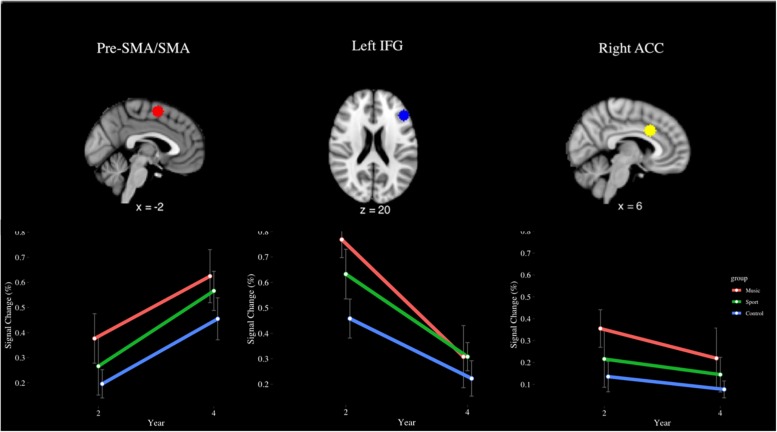
Percent signal change in the pre-SMA/SMA, left IFG, and right ACC during the incongruent > rest contrast of the fMRI Color-Word Stroop Task, by group and year.

The incongruent > rest contrast (see Figure 11) revealed a main effect of year in the SMA [*F*(1,28) = 11.29, *p* < 0.001, η^2^ = 0.19], where participants had greater signal change at Year 4 (*M* = 0.55%) than Year 2 (*M* = 0.28%). In the left IFG, a significant main effect of group was observed [*F*(2,28) = 3.87, *p* < 0.05, η^2^ = 0.10]. This finding indicated the music group (*M* = 0.54%) had greater percent signal change than the control group (0.34%) but was not significant after correcting for multiple comparisons (*p* = 0.08, *d* = 0.59). A main effect of year was also observed [*F*(1,28) = 10.10, *p* < 0.01, η^2^ = 0.28], indicating that percent signal change was greater at year 2 (*M* = 0.62%) than year 4 (*M* = 0.28%). No other significant main or interaction effects were observed for this contrast (*p* > 0.05).

Neither response time nor accuracy of incongruent trials of the Color-Word Stroop task performed outside the scanner, on a separate day, was correlated with Incongruent - Congruent percent signal change in any ROI (*p* > 0.05).

## Discussion

The present study examined the effects of group-based music training on the development of inhibition skills in children from under-resourced communities. Using a longitudinal design, children involved in music training were compared with active and passive comparison groups (children involved in sports training, and no systematic after-school program, respectively). We assessed changes in children’s inhibition skills and its neural correlates over the course of 4 years, using a delayed gratification task, a flanker task, and a Color-Word Stroop task that was performed both inside and outside of an MRI scanner.

We observed gradual improved performance associated with music training in incongruent trials of the flanker task, in which participants were required to inhibit a dominant response. Music participants improved their accuracy significantly in incongruent trials after 3 and 4 years of training, whereas improvement was observed in the sports and control groups but did not reach a level of significant difference. Overall accuracy was not different among groups after 4 years, however. This may be explained by the music group’s slightly, non-significant, lower level of accuracy at Year 2 than the sports and control groups, and that they therefore had more to gain from an intervention. While no group differences were observed at any time point, the development observed in the music group may suggest an effect of training unique to music instruction. This is similar to Holochwost et al.’s (2017) finding that children involved in a community-based orchestra showed improvement in go-no-go and flanker task accuracy after 2 and 3 years of training, and with Jaschke et al.’s (2018) finding of improved go-no-go scores in music participants after 2.5 years of training. However, here, the music group’s improvement becomes evident after slightly later, after 3 years of training, as we did not obtain a baseline measure of this task, we do not know how participants would have performed at an earlier stage of training. Reaction time in the music group was similar to that of the control group, while sports participants showed a non-significant trend of slower response times. This was most apparent at the last year of participation, suggesting an effect related to the length of training on development of inhibition skills. These findings are consistent with previous work citing faster reaction times of child (Joret et al., 2017) and adult (Amer et al., 2013) musicians in comparison to controls in a Simon Arrows Task, where the effect is more pronounced in incongruent trials. These findings are additionally in line with research indicating adult musicians perform with faster reaction times overall than controls on inhibition measures such as Auditory and Color-Word Stroop tasks (Bialystok and Depape, 2009; Travis et al., 2011; Amer et al., 2013).

In the Delayed Gratification task, we observed an accelerated ability to reject a small, immediate reward in favor of a delayed, large reward in children who had been involved in music training in comparison to children who had not been involved in any training. This finding is not explained by other differences among the groups, as all groups were similar with regard to SES, age, and other cognitive tasks. Discounting of future rewards in favor of smaller, immediate rewards declines with age, where children increasingly make rational reward choices as they get older (Bettinger and Slonim, 2007; Steinberg et al., 2009). While the sport and control groups followed this pattern by delaying large rewards by the last year of assessment, the music group did so earlier and more often, demonstrating an ability to rationally delay rewards that was better than expected for their age group. Music-trained children were also less likely to delay discount small rewards as they got older, while sports-trained and control children were more likely to do so. While no other studies to our knowledge have investigated the effect of music training on delayed gratification skills, it is a possibility that attention to detail and orientation to subtle musical cues had a role in extending inhibition to smaller rewards.

Group differences in the delayed gratification and flanker fish tasks, albeit not always statistically significant, indicate a trend toward enhanced inhibition skills in participants involved in music training and are consistent with other longitudinal findings citing overall greater inhibitory control improvements in musically trained children (Moreno et al., 2011; Holochwost et al., 2017; Jaschke et al., 2018). Musicians’ advantages in inhibition skills may relate to paralleled processes in disciplined instrumental practice; learning to play a musical instrument involves frequent stopping to correct mistakes and rehearsing small passages in isolation, which both delays the reward of playing a piece in its entirety and requires one to put aside immediate non-musical distractors for the larger reward of musical proficiency. These skills may additionally relate to musicians’ regular practice of error monitoring, where children playing music must quickly adjust motor behavior in response to unanticipated musical demands and mistakes.

Results from the fMRI Stroop task support the trend observed in the Delayed Gratification task, where children involved in music training demonstrated an accelerated maturity that was not achieved until a later age by the Sports and Control groups. We previously reported that, at Year 2 (after 2 years of music training), music participants as compared to controls showed greater difference in BOLD signal in the cognitive control network in the incongruent versus congruent blocks, specifically in the IFG, pre-SMA/SMA, ACC, precentral gyrus, and insula (Sachs et al., 2017). In the current analysis, after 4 years, we found no differences between groups in this contrast, suggesting that the music group had matured on the task at an earlier age than the control group. In the incongruent > rest contrast, the music group showed greater BOLD signal in the right IFG, a finding not observed at Year 2. While color-word Stroop paradigm has been associated with only left IFG (Taylor et al., 1997; Fan et al., 2003; Bernal and Altman, 2009), some studies have reported bilateral activation (Peterson et al., 1999; Banich et al., 2000; Adleman et al., 2002), often driven by differences in design (event-related vs. block; Leung et al., 2000). The right IFG has instead been linked to the stop-signal task and, specifically, cue detection and attentional monitoring, as opposed to the initiation of a motor response (Aron et al., 2003; Hampshire et al., 2010; Sharp et al., 2010). Given the conflicting literature on the right IFG implicated in the Stroop task, and the relatively small sample size here, we do not draw strong conclusions from this finding. Yet, we note that this may indicate improved inhibitory processing associated with length of music training, where music participants are more strongly marking incongruent stimuli as cues to stop a prepotent response.

Our ROI analysis similarly indicated that significant group differences in percent signal change, when present, occurred at Year 2 but not at Year 4. Such differences at Year 4 were present only as non-significant trends, or as significant effects that did not survive *post hoc* analysis. At Year 2, we found that the music group as compared to both control groups had significantly greater percent signal change in the left IFG, pre-SMA/SMA, and right ACC (Sachs et al., 2017). Here, with a notably smaller sample, we find the group by year interaction term approached significance only for the left IFG. This finding is notable due to the left IFG’s role in inhibiting prepotent motor responses (Swick et al., 2008), which is a skill that is highly practiced by musicians when monitoring their performance. However, the reduced number of participants from Year 2 to 4 may have contributed to the statistically non-significant trends.

It should be noted that several studies report increased engagement of the cognitive control network related to decreased efficiency in inhibition tasks (Tamm et al., 2002; Luna et al., 2010). Others have additionally reported activation decreases in these regions after interventions aimed at improving inhibition skills (Chaddock-Heyman et al., 2013; Nishiguchi et al., 2015). We acknowledge that, without a measure of accuracy inside the scanner, we cannot definitively interpret our BOLD signal findings as indicative of better inhibitory processing. Neural activation observed in the music group at Year 2 thus may have been due to greater cognitive effort required to complete the task and that, by Year 4, all groups were engaging the task with similar levels of effort. However, as accuracy in the Stroop task outside of the scanner trended toward a positive correlation with percent signal change in the pre-SMA/SMA and left IFG at Year 2 (Sachs et al., 2017), our interpretation of these findings aligns more with improved inhibitory control processing.

We note an important and possible confounding factor in this study: the high percentage of bilingualism among participants: 96% of participants retained through Year 4 were Spanish-English bilingual: 100% in the music group, 86.67% in the sports group, and 100% in the control group. Although formal bilingualism measures were not obtained, interview data at baseline indicated that all bilingual participants were fluent in both Spanish and English, primarily speaking Spanish at home and English in the classroom. All bilingual participants indicated that they watched television, read books, and listened to music in both English and Spanish. 66.23% of participants at baseline were enrolled in English as a Second Language classes at their school.

Bilingual individuals have demonstrated advanced inhibitory control skills, as evidenced on performance on the flanker task (Costa et al., 2008; Yang et al., 2011; Barac et al., 2016), stop-signal task (Bialystok et al., 2005; Colzato et al., 2008), Stroop task (Bialystok et al., 2008; Hernández et al., 2010), and Simon task (Martin-Rhee and Bialystok, 2008) when compared to monolingual counterparts, despite lower verbal abilities. These differences are evidenced to emerge as young as 7 months (Kovács and Mehler, 2009), indicating that bilingualism produces enhancements of inhibitory skills before the onset of speech production. Explanations of these findings cite evidence indicating that bilingual individuals activate both languages simultaneously, regardless of context (Hernandez et al., 1996; Costa et al., 1999; Kroll et al., 2008). It has thus been proposed that individuals must constantly suppress the context-dependent irrelevant language in order to communicate effectively, leading to an adaptation in neural attentional networks (Bialystok et al., 2009; Green and Abutalebi, 2013). Studies investigating functional brain organization indicate that bilinguals recruit the frontal regions typically associated with executive control, such as the DLPFC, when switching between languages (Hernandez et al., 2000; Hernandez, 2009; Luk et al., 2011). Despite these findings, a recent meta-analysis reported no evidence for a bilingual advantage in executive functioning in adults (Lehtonen et al., 2018), yet did not investigate studies involving children.

Music training and bilingualism may share similar mechanisms in relation to developing inhibitory control skills. While bilingualism and music playing both require the suppression of irrelevant stimuli, additional similarities exist at a neural level. Musicians, in comparison to musically un-trained individuals, activate the DLPFC when passively listening to music (Ohnishi et al., 2001), as bilinguals do when switching between languages (i.e., Hernandez et al., 2000). Koelsch et al. (2005) observed that, when listening to irregular versus regular musical chords, adults and children engage frontal regions, including the inferior frontal gyrus, and that this activity was positively correlated with music training in both age groups. Thus, both musicians and bilinguals appear to recruit regions associated with executive control when engaging in their respective domains, suggesting that intensive experience in either activity would contribute to enhanced inhibition skills.

Given the above evidence, and the fact that almost all of the participants in this study are bilingual, one possible explanation for discrepancies between our results and what we expected to be the effect of music training on inhibition is the contribution of bilingualism. While music training may have an effect, it is likely that multiple factors, including bilingualism, benefit inhibitory control skills. In studies comparing the effects of music training and bilingualism, bilinguals have been reported to perform similarly (Bialystok and Depape, 2009; Schroeder et al., 2016) or better than (Janus et al., 2016) musically trained individuals on measures of inhibition. Other research has indicated no interaction between music training and bilingualism on measures of inhibition, suggesting that both factors may independently contribute to the development of inhibitory control, but do not produce additional combined effects (Moradzadeh et al., 2015; Schroeder et al., 2016). A proposed explanation for the lack of additive benefits of music training and bilingualism in these studies was that each factor alone produced ceiling effects, restricting further benefit of the combination of experiences. Here, we observe differences, while not always significant, between groups in which nearly all participants are bilingual, suggesting that music training and bilingualism may have a small additive effect early in development. We do, however, acknowledge that, if Lehtonen et al. (2018)’s findings generalize to children, the possible explanation related to bilingualism may not be relevant in our findings.

A limitation of the present study is the inevitable decrease of sample size from baseline to Year 4. While we took preventive measures to reduce attrition, it is typical for longitudinal studies, in particular with a population from underrepresented communities, to experience a reduction in participant number over time. However, attrition was similar in all groups. Additionally, as this is not a randomized control trial, it is possible that our participants had pre-existing differences for which we could not account, such as home environment and parental motivation between groups. A strength of this study is that participants were assessed at baseline and demonstrated no differences between groups in measures of musical pitch and rhythm discrimination (Ilari et al., 2016); it is nonetheless possible that children with inherent musical motivation persisted in the music training program. However, the absence of group differences at baseline in other key variables strongly suggests that any observed enhancements of inhibitory control are due to the intervention in contrast to pre-existing factors.

Despite these limitations, we provide evidence that 4 years of group-based music training leads to modest positive effects on inhibition skills, evidenced by a greater rate of accuracy improvement on a Flanker task and increased rational decisions on a Delayed Gratification task, and its neural correlates in children from underserved communities. The absence of consistent group differences across all inhibition measures point to the other possible explanations such as bilingualism. When present, advantages associated with music training manifest as early, as musicians demonstrate accelerated development of inhibition, but later diminish as non-musically trained children catch up in these skills.

## Data Availability Statement

The datasets generated for this study are available on request to the corresponding author.

## Ethics Statement

The studies involving human participants were reviewed and approved by University of Southern California Institutional Review Board. Written informed consent to participate in this study was provided by the participants’ legal guardian/next of kin.

## Author Contributions

AH and BI: conceptualization and supervision. SH, BI, and AH: data curation. SH and AH: behavioral analysis and wrote the manuscript. SH and MS: fMRI analysis and visualization. AH: funding acquisition, investigation, resources, and software. SH: project administration.

## Conflict of Interest

The authors declare that the research was conducted in the absence of any commercial or financial relationships that could be construed as a potential conflict of interest.

## References

[B1] Adleman N. E. Menon V. Blasey C. M. White C. D. Warsofsky I. S. Glover G. H. (2002). A developmental fMRI study of the stroop color-word task. *Neuroimage* 16 61–75. 10.1006/nimg.2001.1046 11969318

[B2] Allen M. Dietz M. Blair K. S. van Beek M. Rees G. Vestergaard-Poulsen P. (2012). Cognitive-affective neural plasticity following active-controlled mindfulness intervention. *J. Neurosci.* 32 15601–15610. 10.1523/JNEUROSCI.2957-12.2012 23115195 PMC4569704

[B3] Alloway T. P. Gathercole S. E. Adams A. M. Willis C. Eaglen R. Lamont E. (2005). Working memory and phonological awareness as predictors of progress towards early learning goals at school entry. *Br. J. Dev. Psychol.* 23 417–426. 10.1348/026151005x26804

[B4] Amer T. Kalender B. Hasher L. Trehub S. E. Wong Y. (2013). Do older professional musicians have cognitive advantages? *PLoS One* 8:e71630. 10.1371/journal.pone.0071630 23940774 PMC3737101

[B5] Aron A. R. Fletcher P. C. Bullmore E. T. Sahakian B. J. Robbins T. W. (2003). Stop-signal inhibition disrupted by damage to right inferior frontal gyrus in humans. *Nat. Neurosci.* 6 115–116. 10.1038/nn1003 12536210

[B6] Bailey C. E. (2007). Cognitive accuracy and intelligent executive function in the brain and in business. *Ann. N. Y. Acad. Sci.* 1118 122–141. 10.1196/annals.1412.011 17717092

[B7] Banich M. T. Milham M. P. Atchley R. Cohen N. J. Webb A. Wszalek T. (2000). fMRI studies of stroop tasks reveal unique roles of anterior and posterior brain systems in attentional selection. *J. Cogn. Neurosci.* 12 988–1000. 10.1162/08989290051137521 11177419

[B8] Barac R. Moreno S. Bialystok E. (2016). Behavioral and electrophysiological differences in executive control between monolingual and bilingual children. *Child Dev.* 87 1277–1290. 10.1111/cdev.12538 27133956 PMC4939124

[B9] Berkman E. T. Kahn L. E. Merchant J. S. (2014). Training-induced changes in inhibitory control network activity. *J. Neurosci.* 34 149–157. 10.1523/jneurosci.3564-13.2014 24381276 PMC3866481

[B10] Bernal B. Altman N. (2009). Neural networks of motor and cognitive inhibition are dissociated between brain hemispheres: an fMRI study. *Int. J. Neurosci.* 119 1848–1880. 10.1080/00207450802333029 19922390

[B11] Bettinger E. Slonim R. (2007). Patience among children. *J. Publ. Econ.* 91 343–363. 10.1016/j.jpubeco.2006.05.010

[B12] Bialystok E. Craik F. Luk G. (2008). Cognitive control and lexical access in younger and older bilinguals. *J. Exp. Psychol. Learn. Mem. Cogn.* 34 859–873. 10.1037/0278-7393.34.4.859 18605874

[B13] Bialystok E. Craik F. I. Grady C. Chau W. Ishii R. Gunji A. (2005). Effect of bilingualism on cognitive control in the simon task: evidence from MEG. *NeuroImage* 24 40–49. 10.1016/j.neuroimage.2004.09.044 15588595

[B14] Bialystok E. Craik F. I. Green D. W. Gollan T. H. (2009). Bilingual minds. *Psychol. Sci. Publ. Interest* 10 89–129. 10.1177/1529100610387084 26168404

[B15] Bialystok E. Depape A. M. (2009). Musical expertise, bilingualism, and executive functioning. *J. Exp. Psychol. Hum. Percept. Perform.* 35 565–574. 10.1037/a0012735 19331508

[B16] Brainard D. H. (1997). The psychophysics toolbox. *Spat. Vis.* 10 433–436. 10.1163/156856897x00357 9176952

[B17] Chaddock-Heyman L. Erickson K. I. Voss M. Knecht A. Pontifex M. B. Castelli D. (2013). The effects of physical activity on functional MRI activation associated with cognitive control in children: a randomized controlled intervention. *Front. Hum. Neurosci.* 7:72. 10.3389/fnhum.2013.00072 23487583 PMC3594762

[B18] Chang Y. K. Liu S. Yu H. H. Lee Y. H. (2012). Effect of acute exercise on executive function in children with attention deficit hyperactivity disorder. *Arch. Clin. Neuropsychol.* 27 225–237. 10.1093/arclin/acr094 22306962

[B19] Chen A. G. Yan J. Yin H. C. Pan C. Y. Chang Y. K. (2014). Effects of acute aerobic exercise on multiple aspects of executive function in preadolescent children. *Psychol. Sport Exerc.* 15 627–636. 10.1016/j.psychsport.2014.06.004

[B20] Colzato L. S. Bajo M. T. van den Wildenberg W. Paolieri D. Nieuwenhuis S. La Heij W. (2008). How does bilingualism improve executive control? A comparison of active and reactive inhibition mechanisms. *J. Exp. Psychol. Learn. Mem. Cogn.* 34 302–312. 10.1037/0278-7393.34.2.302 18315407

[B21] Costa A. Hernández M. Sebastián-Gallés N. (2008). Bilingualism aids conflict resolution: evidence from the ANT task. *Cognition* 106 59–86. 10.1016/j.cognition.2006.12.013 17275801

[B22] Costa A. Miozzo M. Caramazza A. (1999). Lexical selection in bilinguals: do words in the bilingual’s two lexicons compete for selection? *J. Mem. Langu.* 41 365–397. 10.1006/jmla.1999.2651

[B23] Davidson M. C. Amso D. Anderson L. C. Diamond A. (2006). Development of cognitive control and executive functions from 4 to 13 years: evidence from manipulations of memory, inhibition, and task switching. *Neuropsychologia* 44 2037–2078. 10.1016/j.neuropsychologia.2006.02.006 16580701 PMC1513793

[B24] Davis C. L. Tomporowski P. D. McDowell J. E. Austin B. P. Miller P. H. Yanasak N. E. (2011). Exercise improves executive function and achievement and alters brain activation in overweight children: a randomized, controlled trial. *Health Psychol.* 30 91–98. 10.1037/a0021766 21299297 PMC3057917

[B25] Degé F. Schwarzer G. (2011). The effect of a music program on phonological awareness in preschoolers. *Front. Psychol.* 2:124. 10.3389/fpsyg.2011.00124 21734895 PMC3121007

[B26] Diamond A. Barnett W. S. Thomas J. Munro S. (2007). Preschool program improves cognitive control. *Science* 318 1387–1388. 10.1126/science.1151148 18048670 PMC2174918

[B27] Duckworth A. L. Seligman M. E. (2005). Self-discipline outdoes IQ in predicting academic performance of adolescents. *Psychol. Sci.* 16 939–944. 10.1111/j.1467-9280.2005.01641.x 16313657

[B28] Eisenberg N. Sadovsky A. Spinrad T. L. Fabes R. A. Losoya S. H. Valiente C. (2005). The relations of problem behavior status to children’s negative emotionality, effortful control, and impulsivity: concurrent relations and prediction of change. *Dev. Psychol.* 41 193–211. 10.1037/0012-1649.41.1.193 15656749 PMC1361290

[B29] Fan J. Flombaum J. I. McCandliss B. D. Thomas K. M. Posner M. I. (2003). Cognitive and brain consequences of conflict. *Neuroimage* 18 42–57. 10.1006/nimg.2002.1319 12507442

[B30] Flook L. Smalley S. L. Kitil M. J. Galla B. M. Kaiser-Greenland S. Locke J. (2010). Effects of mindful awareness practices on executive functions in elementary school children. *J. Appl. Sch. Psychol.* 26 70–95. 10.1080/15377900903379125

[B31] Green D. W. Abutalebi J. (2013). Language control in bilinguals: the adaptive control hypothesis. *J. Cogn. Psychol.* 25 515–530. 10.1080/20445911.2013.796377 25077013 PMC4095950

[B32] Habibi A. Damasio A. Ilari B. Veiga R. Joshi A. A. Leahy R. M. (2017). Childhood music training induces change in micro and macroscopic brain structure: results from a longitudinal study. *Cereb. Cortex* 28 4336–4347. 10.1093/cercor/bhx286 29126181

[B33] Habibi A. Ilari B. Crimi K. Metke M. Kaplan J. T. Joshi A. A. (2014). An equal start: absence of group differences in cognitive, social, and neural measures prior to music or sports training in children. *Front. Hum. Neurosci.* 8:690. 10.3389/fnhum.2014.00690 25249961 PMC4158792

[B34] Hampshire A. Chamberlain S. R. Monti M. M. Duncan J. Owen A. M. (2010). The role of the right inferior frontal gyrus: inhibition and attentional control. *Neuroimage* 50 1313–1319. 10.1016/j.neuroimage.2009.12.109 20056157 PMC2845804

[B35] Henrich J. Heine S. J. Norenzayan A. (2010). The weirdest people in the world? *Behav. Brain Sci.* 33 61–83. 10.1017/s0140525x0999152x 20550733

[B36] Hernandez A. E. (2009). Language switching in the bilingual brain: what’s next? *Brain Lang.* 109 133–140. 10.1016/j.bandl.2008.12.005 19250662

[B37] Hernandez A. E. Bates E. A. Avila L. X. (1996). Processing across the language boundary: a cross-modal priming study of Spanish-English bilinguals. *J. Exp. Psychol. Learn. Mem. Cogn.* 22 846–864. 10.1037//0278-7393.22.4.846 8708603

[B38] Hernandez A. E. Martinez A. Kohnert K. (2000). In search of the language switch: an fMRI study of picture naming in Spanish-English bilinguals. *Brain Lang.* 73 421–431. 10.1006/brln.1999.2278 10860563

[B39] Hernández M. Costa A. Fuentes L. J. Vivas A. B. Sebastián-Gallés N. (2010). The impact of bilingualism on the executive control and orienting networks of attention. *Bilingualism* 13 315–325. 10.1017/s1366728909990010

[B40] Holochwost S. J. Propper C. B. Wolf D. P. Willoughby M. T. Fisher K. R. Kolacz J. (2017). Music education, academic achievement, and executive functions. *Psychol. Aesthet. Creat. Arts* 11 147–166. 10.1037/aca0000112

[B41] Hughes C. Dunn J. (1998). Understanding mind and emotion: longitudinal associations with mental-state talk between young friends. *Dev. Psychol.* 34 1026–1037. 10.1037//0012-1649.34.5.1026 9779748

[B42] Ilari B. S. Keller P. Damasio H. Habibi A. (2016). The development of musical skills of underprivileged children over the course of 1 year: a study in the context of an El Sistema-inspired program. *Front. Psychol.* 7:62. 10.3389/fpsyg.2016.00062 26869964 PMC4735430

[B43] Janus M. Lee Y. Moreno S. Bialystok E. (2016). Effects of short-term music and second-language training on executive control. *J. Exp. Child Psychol.* 144 84–97. 10.1016/j.jecp.2015.11.009 26709746 PMC4724315

[B44] Jaschke A. C. Honing H. Scherder E. J. (2018). Longitudinal analysis of music education on executive functions in primary school children. *Front. Neurosci.* 12:103. 10.3389/fnins.2018.00103 29541017 PMC5835523

[B45] Jentzsch I. Mkrtchian A. Kansal N. (2014). Improved effectiveness of performance monitoring in amateur instrumental musicians. *Neuropsychologia* 52 117–124. 10.1016/j.neuropsychologia.2013.09.025 24056298 PMC3905185

[B46] Joret M. E. Germeys F. Gidron Y. (2017). Cognitive inhibitory control in children following early childhood music education. *Music. Sci.* 21 303–315. 10.1177/1029864916655477

[B47] Keller P. E. (2008). “Joint action in music performance,” in *Emerging Communication: Studies on New Technologies and Practices in Communication: Enacting Intersubjectivity: A Cognitive and Social Perspective on the Study of Interactions*, Vol. 10 eds Morganti F. Carassa A. Riva G. (Amsterdam: IOS Press), 205–221.

[B48] Kirby K. N. Winston G. C. Santiesteban M. (2005). Impatience and grades: delay-discount rates correlate negatively with college GPA. *Learn. Individ. Diff.* 15 213–222. 10.1016/j.lindif.2005.01.003

[B49] Koelsch S. Fritz T. Schulze K. Alsop D. Schlaug G. (2005). Adults and children processing music: an fMRI study. *Neuroimage* 25 1068–1076. 10.1016/j.neuroimage.2004.12.050 15850725

[B50] Kovács ÁM. Mehler J. (2009). Cognitive gains in 7-month-old bilingual infants. *Proc. Natl. Acad. Sci. U.S.A.* 106 6556–6560. 10.1073/pnas.0811323106 19365071 PMC2672482

[B51] Kroll J. F. Bobb S. C. Misra M. Guo T. (2008). Language selection in bilingual speech: evidence for inhibitory processes. *Acta Psychol.* 128 416–430. 10.1016/j.actpsy.2008.02.001 18358449 PMC2585366

[B52] Lakes K. D. Hoyt W. T. (2004). Promoting self-regulation through school-based martial arts training. *J. Appl. Dev. Psychol.* 25 283–302. 10.1016/j.appdev.2004.04.002

[B53] Lehtonen M. Soveri A. Laine A. Järvenpää J. de Bruin A. Antfolk J. (2018). Is bilingualism associated with enhanced executive functioning in adults? A meta-analytic review. *Psychol. Bull.* 144 394–425. 10.1037/bul0000142 29494195

[B54] Lengua L. J. (2003). Associations among emotionality, self-regulation, adjustment problems, and positive adjustment in middle childhood. *J. Appl. Dev. Psychol.* 24 595–618. 10.1016/j.appdev.2003.08.002 PMC2777689 20046898

[B55] Leung H. C. Skudlarski P. Gatenby J. C. Peterson B. S. Gore J. C. (2000). An event-related functional MRI study of the Stroop color word interference task. *Cereb. Cortex* 10 552–560. 10.1093/cercor/10.6.552 10859133

[B56] Luk G. Green D. W. Abutalebi J. Grady C. (2011). Cognitive control for language switching in bilinguals: a quantitative meta-analysis of functional neuroimaging studies. *Lang. Cogn. Process.* 27 1479–1488. 10.1080/01690965.2011.613209 24795491 PMC4006828

[B57] Luna B. Padmanabhan A. O’Hearn K. (2010). What has fMRI told us about the development of cognitive control through adolescence? *Brain Cogn.* 72 101–113. 10.1016/j.bandc.2009.08.005 19765880 PMC2815087

[B58] Martin-Rhee M. M. Bialystok E. (2008). The development of two types of inhibitory control in monolingual and bilingual children. *Bilingualism* 11 81–93. 10.1017/s1366728907003227 25935936

[B59] Miller E. K. Cohen J. D. (2001). An integrative theory of prefrontal cortex function. *Annu. Rev. Neurosci.* 24 167–202. 10.1146/annurev.neuro.24.1.167 11283309

[B60] Miller H. V. Barnes J. C. Beaver K. M. (2011). Self-control and health outcomes in a nationally representative sample. *Am. J. Health Behav.* 35 15–27. 20950155 10.5993/ajhb.35.1.2

[B61] Mischel W. Shoda Y. Rodriguez M. I. (1989). Delay of gratification in children. *Science* 244 933–938. 10.1126/science.2658056 2658056

[B62] Miyake A. Friedman N. P. Emerson M. J. Witzki A. H. Howerter A. Wager T. D. (2000). The unity and diversity of executive functions and their contributions to complex “frontal lobe” tasks: a latent variable analysis. *Cogn. Psychol.* 41 49–100. 10.1006/cogp.1999.0734 10945922

[B63] Moffitt T. E. Arseneault L. Belsky D. Dickson N. Hancox R. J. Harrington H. (2011). A gradient of childhood self-control predicts health, wealth, and public safety. *Proc. Natl. Acad. Sci. U.S.A.* 108 2693–2698. 10.1073/pnas.1010076108 21262822 PMC3041102

[B64] Moradzadeh L. Blumenthal G. Wiseheart M. (2015). Musical training, bilingualism, and executive function: a closer look at task switching and dual-task performance. *Cogn. Sci.* 39 992–1020. 10.1111/cogs.12183 25289704

[B65] Moreno S. Bialystok E. Barac R. Schellenberg E. G. Cepeda N. J. Chau T. (2011). Short-term music training enhances verbal intelligence and executive function. *Psychol. Sci.* 22 1425–1433. 10.1177/0956797611416999 21969312 PMC3449320

[B66] Münte T. F. Altenmüller E. Jäncke L. (2002). The musician’s brain as a model of neuroplasticity. *Nat. Rev. Neurosci.* 3 473–478. 12042882 10.1038/nrn843

[B67] Niendam T. A. Laird A. R. Ray K. L. Dean Y. M. Glahn D. C. Carter C. S. (2012). Meta-analytic evidence for a superordinate cognitive control network subserving diverse executive functions. *Cogn. Affect. Behav. Neurosci.* 12 241–268. 10.3758/s13415-011-0083-5 22282036 PMC3660731

[B68] Nishiguchi S. Yamada M. Tanigawa T. Sekiyama K. Kawagoe T. Suzuki M. (2015). A 12-week physical and cognitive exercise program can improve cognitive function and neural efficiency in community-dwelling older adults: a randomized controlled trial. *J. Am. Geriatr. Soc.* 63 1355–1363. 10.1111/jgs.13481 26114906

[B69] Ohnishi T. Matsuda H. Asada T. Aruga M. Hirakata M. Nishikawa M. (2001). Functional anatomy of musical perception in musicians. *Cereb. Cortex* 11 754–760. 10.1093/cercor/11.8.754 11459765

[B70] Peterson B. S. Skudlarski P. Gatenby J. C. Zhang H. Anderson A. W. Gore J. C. (1999). An fMRI study of Stroop word-color interference: evidence for cingulate subregions subserving multiple distributed attentional systems. *Biol. Psychiatry* 45 1237–1258. 10.1016/s0006-3223(99)00056-6 10349031

[B71] Power J. D. Barnes K. A. Snyder A. Z. Schlaggar B. L. Petersen S. E. (2012). Spurious but systematic correlations in functional connectivity MRI networks arise from subject motion. *Neuroimage* 59 2142–2154. 10.1016/j.neuroimage.2011.10.018 22019881 PMC3254728

[B72] R Core Team (2018). *R: A Language and Environment for Statistical Computing.* Vienna: R Foundation for Statistical Computing.

[B73] Saarikivi K. Putkinen V. Tervaniemi M. Huotilainen M. (2016). Cognitive flexibility modulates maturation and music-training-related changes in neural sound discrimination. *Eur. J. Neurosci.* 44 1815–1825. 10.1111/ejn.13176 26797826

[B74] Sachs M. Kaplan J. Der Sarkissian A. Habibi A. (2017). Increased engagement of the cognitive control network associated with music training in children during an fMRI Stroop task. *PLoS One* 12:e0187254. 10.1371/journal.pone.0187254 29084283 PMC5662181

[B75] Schellenberg E. G. (2011). Examining the association between music lessons and intelligence. *Br. J. Psychol.* 102 283–302. 10.1111/j.2044-8295.2010.02000.x 21751987

[B76] Schroeder S. R. Marian V. Shook A. Bartolotti J. (2016). Bilingualism and musicianship enhance cognitive control. *Neural Plast.* 2016:4058620. 10.1155/2016/4058620 26819764 PMC4706931

[B77] Seeyave D. M. Coleman S. Appugliese D. Corwyn R. F. Bradley R. H. Davidson N. S. (2009). Ability to delay gratification at age 4 years and risk of overweight at age 11 years. *Arch. Pediatr. Adolesc. Med.* 163 303–308. 10.1001/archpediatrics.2009.12 19349558 PMC4174459

[B78] Sharp D. J. Bonnelle V. De Boissezon X. Beckmann C. F. James S. G. Patel M. C. (2010). Distinct frontal systems for response inhibition, attentional capture, and error processing. *Proc. Natl. Acad. Sci. U.S.A.* 107 6106–6111. 10.1073/pnas.1000175107 20220100 PMC2851908

[B79] Slevc L. R. Davey N. S. Buschkuehl M. Jaeggi S. M. (2016). Tuning the mind: exploring the connections between musical ability and executive functions. *Cognition* 152 199–211. 10.1016/j.cognition.2016.03.017 27107499

[B80] Steinberg L. Graham S. O’Brien L. Woolard J. Cauffman E. Banich M. (2009). Age differences in future orientation and delay discounting. *Child Dev.* 80 28–44. 10.1111/j.1467-8624.2008.01244.x 19236391

[B81] Swick D. Ashley V. Turken U. (2008). Left inferior frontal gyrus is critical for response inhibition. *BMC Neurosci.* 9:102. 10.1186/1471-2202-9-102 18939997 PMC2588614

[B82] Tamm L. Menon V. Reiss A. L. (2002). Maturation of brain function associated with response inhibition. *J. Am. Acad. Child Adolesc. Psychiatry* 41 1231–1238. 10.1097/00004583-200210000-00013 12364845

[B83] Taylor S. F. Kornblum S. Lauber E. J. Minoshima S. Koeppe R. A. (1997). Isolation of specific interference processing in the Stroop task: PET activation studies. *Neuroimage* 6 81–92. 10.1006/nimg.1997.0285 9299382

[B84] Travis F. Harung H. S. Lagrosen Y. (2011). Moral development, executive functioning, peak experiences and brain patterns in professional and amateur classical musicians: interpreted in light of a Unified Theory of Performance. *Conscious. Cogn.* 20 1256–1264. 10.1016/j.concog.2011.03.020 21507681

[B85] Voss M. W. Chaddock L. Kim J. S. VanPatter M. Pontifex M. B. Raine L. B. (2011). Aerobic fitness is associated with greater efficiency of the network underlying cognitive control in preadolescent children. *Neuroscience* 199 166–176. 10.1016/j.neuroscience.2011.10.009 22027235 PMC3237764

[B86] Wechsler D. (2011). *Wechsler Abbreviated Scale of Intelligence*, 2nd Edn Bloomington, MN: Pearson.

[B87] Wright I. Waterman M. Prescott H. Murdoch-Eaton D. (2003). A new Stroop-like measure of inhibitory function development: typical developmental trends. *J. Child Psychol. Psychiatry* 44 561–575. 10.1111/1469-7610.00145 12751848

[B88] Yang S. Yang H. Lust B. (2011). Early childhood bilingualism leads to advances in executive attention: dissociating culture and language. *Bilingualism* 14 412–422. 10.1017/s1366728910000611

[B89] Zatorre R. J. Chen J. L. Penhune V. B. (2007). When the brain plays music: auditory–motor interactions in music perception and production. *Nat. Rev. Neurosci.* 8 547–558. 10.1038/nrn2152 17585307

[B90] Zuk J. Benjamin C. Kenyon A. Gaab N. (2014). Behavioral and neural correlates of executive functioning in musicians and non-musicians. *PLoS One* 9:e99868. 10.1371/journal.pone.0099868 24937544 PMC4061064

