# Supplementary material for: Effects of Music Training on Inhibitory Control and Associated Neural Networks in School-Aged Children: A Longitudinal Study
Source: Front Neurosci. 2019 Oct 16;13:1080. doi: 10.3389/fnins.2019.01080 (PMC6805726; doi:10.3389/fnins.2019.01080)
Supplement: Supplementary file 1 [file Data_Sheet_1.docx]

Supplementary Material

# Assessment of cognitive development

Cognitive development was assessed using the Wechsler Abbreviated Scale of Intelligences (WASI-II) for children (Wechsler, 1999). Four subtests were administered: Block Design, Vocabulary, Matrix Reasoning, and Similarities. In the Block Design subtest, the participant was shown a picture of an abstract design and was instructed to recreate the design using a set of red and white blocks as fast as possible. Participants were timed and continued until two designs in a row were created incorrectly or not within the time limit. In the vocabulary subtest, the participant was asked to define words read aloud. In the Matrix Reasoning subtest, participants were asked to select an image from four to six pictures that best fit a design with a missing space. In the Similarities subtest, children were asked to describe how two dictated words are similar. In the latter three subtests, participants were not timed and continued until three items received a score of zero.

# Assessment of inhibition

## Flanker Fish Task

The Flanker Fish Task (Davidson et al., 2006; Diamond et al., 2007) is a child-friendly version of the Flanker task, administered starting Year 2 (Figure 2). Participants were presented an image of seven fish in a row and were asked to press the response button on the same side that the middle fish is facing. Non-target fish were either all facing the same direction as the target (congruent condition), the opposite direction as the target (incongruent condition), or neither the same nor opposite direction as the target (neutral condition). In the neutral condition, non-target fish were either facing up or down, or were not present at all. The task consisted of 17 trials total, and participants had 1500 ms to respond. Untimed practice rounds (5 trials each) were presented, during which the participant was unable to advance to the next question until they had responded correctly. Stimuli were presented on a HP laptop computer using Presentation ® software (Version 18.2, Neurobehavioral Systems, Inc., Berkeley, CA, www.neurobs.com).

## Delayed Gratification Task

A version of the delay task (Mischel et al., 1989) was administered starting Year 3. Participants were physically presented with six sets of rewards of either M&Ms or quarters, and told that they could choose to have the reward now or at their next visit, within a week. Participants were told that there was no right or wrong answer, and their choice was entirely their decision. Rewards were either small (1 now, 2 later), medium (1 now, 4 later), or large (1 now, 6 later) and were equally divided between M&Ms and quarters. Sets were randomized between participants.

## Stroop Task

A child-friendly version of the Stroop task was assessed Year 1 (Wright et al., 2003). Participants were asked to verbally identify the name of an animal based on its body. In the congruent condition, the animal head matched the body (24 trials total). In the incongruent condition, the animal head did not match the body (24 trials total). Trials were administered via paper sheets, and examiners recorded verbal responses.

Starting Year 2, a Color-Word Stroop task was administered. Participants were instructed to identify the color of the ink of a presented word using keys on a keyboard as quickly and as accurately as possible. The participant completed six blocks (12 trials) of the task. Each block consisted of either all congruent trials, in which the color of the word matched the written word (e.g: “blue” written in blue ink), or all incongruent trials, in which the color of the word did not match the written word (e.g: “blue” written in yellow ink). Ink colors appeared exactly three times in each block, but there were no restrictions on the number of times the written color was presented. Trials within blocks were randomly permutated to create two unique randomizations (“A” and “B”). Years 2 and 3, participants reported answers verbally. Year 4, participants reported answers with keyboard presses corresponding to different colors. Participants were given 1700ms to respond. Before beginning the task, participants engaged in two training sessions; the first, to learn the keyboard letters corresponding to four different presented colors, and the second, to practice the color-word identification in an untimed setting. During training, the participant was unable to advance to the next trial until they had responded correctly. Stimuli were presented on a MacBook laptop computer using Matlab (Mathworks, Natick, MA, USA) and the Psychophysics Toolbox extension (Brainard, 1997).

## fMRI Stroop Task

An fMRI version of the Color-Word Stroop task was assessed inside the scanner at Year 2 and Year 4. Children were presented with words written in one of four colors (red, blue, yellow, or green) and were asked to press a button and to subvocalize the color of the letters as quickly and as accurately as possible. Participants were asked to subvocalize, rather than to voice their responses out loud, to minimize motion artifacts. While subvocalization makes it difficult to determine if individuals are performing the task correctly, participants underwent extensive pre-scanning training to ensure proper understanding of the task. Participants additionally were instructed to press a button after each subvocalization to ensure they are engaged in the task. During training, participants were presented with three blocks (1 congruent, 2 incongruent), with four stimuli in each. During scanning, participants completed two functional runs of six blocks each (3 congruent, 3 incongruent). Each block consisted of 12 trials, with 1700 ms to respond to the stimuli, and 300ms between each trial. Each block was followed by a 16 second rest period. Total scan time was 240 seconds (120 TRs).

# Supplementary Tables

|  | Effect | df | F | p | η^2^ |
| --- | --- | --- | --- | --- | --- |
| **Cognitive Development** |  |  |  |  |  |
| **WASI** |  |  |  |  |  |
| VCI |  |  |  |  |  |
|  | Group | (2, 49) | 2.22 | 0.12 | 0.06 |
|  | Year | (4, 196) | 1.86 | 0.12 | 0.01 |
|  | Year*Group | (8, 196) | 1.20 | 0.30 | 0.01 |
| PRI |  |  |  |  |  |
|  | Group | (2, 49) | 3.14 | 0.05 | 0.09 |
|  | Year | (4, 196) | 2.65* | 0.03 | 0.01 |
|  | Year*Group | (8, 196) | 1.62 | .12 | 0.02 |
| FSIQ-2 |  |  |  |  |  |
|  | Group | (2, 49) | 1.92 | 0.16 | 0.05 |
|  | Year | (4, 196) | 1.59 | 0.18 | 0.01 |
|  | Year*Group | (2, 196) | 1.72 | 0.10 | 0.02 |
| FSIQ-4 |  |  |  |  |  |
|  | Group | (2, 49) | 1.22 | 0.30 | 0.04 |
|  | Year | (4, 196) | 2.33 | 0.06 | 0.01 |
|  | Year*Group | (8, 196) | 1.23 | 0.29 | 0.01 |
| **Inhibition** |  |  |  |  |  |
| **Delayed Gratification** |  |  |  |  |  |
| Quarters |  |  |  |  |  |
|  | Group | (2, 56) | 2.08 | 0.14 | 0.07 |
|  | Year | (1, 56) | 0.01 | 0.93 | 0.00 |
|  | Reward Size | (1.72, 96.2)† | 4.88** | 0.01 | 0.08 |
|  | Year*Group | (2, 56) | 0.62 | 0.54 | 0.02 |
|  | Reward Size*Group | (3.44, 96.20)† | 1.12 | 0.35 | 0.04 |
|  | Year*Reward Size | (1.65, 92.47)† | 7.21*** | 0.001 | 0.14 |
|  | Year*Reward Size*Group | (3.30, 92,47)† | 0.50 | 0.74 | 0.02 |
| M&Ms |  |  |  |  |  |
|  | Group | (2, 56) | 1.58 | 0.22 | 0.00 |
|  | Year | (1, 56) | 0.06 | 0.81 | 0.00 |
|  | Reward Size | (2, 112) | 15.42**** | 0.00 | 0.22 |
|  | Year*Group | (2, 56) | 0.23 | 0.80 | 0.01 |
|  | Reward Size*Group | (4, 112) | 0.39 | 0.82 | 0.01 |
|  | Year*Reward Size | (1.78, 99.81)† | 1.46 | 0.24 | 0.03 |
|  | Year*Reward Size*Group | (3.57, 99.81)† | 2.80** | 0.03 | 0.09 |
| **Animal Stroop** |  |  |  |  |  |
| Reaction Time | Group | (2, 51) | 0.08 | 0.92 | 0.00 |
| Errors | Group | (2, 51) | 0.73 | 0.49 | 0.03 |
| **Color-Word Stroop (Y2/Y3)** |  |  |  |  |  |
| Accuracy | Group | (2, 39) | 0.04 | 0.96 | 0.00 |
|  | Year | (1,39) | 11.96**** | 0.00 | 0.24 |
|  | Condition | (1, 39) | 36.74**** | 0.00 | 0.49 |
|  | Year*Group | (2, 39) | 0.06 | 0.95 | 0.15 |
|  | Condition*Group | (2, 39) | 0.56 | 0.58 | 0.03 |
|  | Year*Condition*Group | (2, 39) | 0.58 | 0.57 | 0.03 |
| Reaction Time | Group | (2, 40) | 0.84 | 0.44 | 0.04 |
|  | Year | (1, 40) | 0.58 | 0.45 | 0.01 |
|  | Year*Group | (2, 40) | 0.88 | 0.88 | 0.01 |
| **Color-Word Stroop (Y4)** |  |  |  |  |  |
| Accuracy | Group | (2, 40) | 0.68 | 0.51 | 0.03 |
|  | Condition | (1, 40) | 22.27**** | 0.00 | 0.36 |
|  | Condition*Group | (2, 40) | 1.44* | 0.07 | 0.07 |
| Reaction Time | Group | (2, 40) | 0.84 | 0.44 | 0.04 |
|  | Condition | (1,40) | 49.77**** | 0.00 | 0.55 |
|  | Condition*Group | (2, 40) | 1.45 | 0.25 | 0.07 |
| **Flanker Fish** |  |  |  |  |  |
| Accuracy | Group | (2, 45) | 0.05 | 0.95 | 0.00 |
|  | Year | (1.17, 52.68)† | 7.02** | 0.03 | 0.14 |
|  | Condition | (1.54, 69.45)† | 13.58**** | 0.00 | 0.23 |
|  | Year*Group | (4, 90) | 0.32 | 0.28 | 0.01 |
|  | Condition*Group | (4, 90) | 0.11 | 0.97 | 0.01 |
|  | Year*Condition | (2.93, 131.81)† | 1.97 | 0.16 | 0.04 |
|  | Year*Condition*Group | (8, 180) | 2.061 | 0.25 | 0.08 |
| Reaction Time | Group | (2, 45) | 2.98* | 0.06 | 0.12 |
|  | Year | (2, 90) | 41.43**** | 0.00 | 0.48 |
|  | Condition | (1.77, 79.47)† | 189.40**** | 0.00 | 0.81 |
|  | Year*Group | (4, 90) | 0.60 | 0.66 | 0.03 |
|  | Condition*Group | (4, 90) | 2.41* | 0.06 | 0.10 |
|  | Year*Condition | (4, 90) | 4.56**** | 0.00 | 0.92 |
|  | Year*Condition*Group | (8, 180) | 0.28 | 0.97 | 0.01 |
|  |  |  |  |  |  |

**Supplementary Table 1.** Behavioral Results.
